# Bone loss in aseptic revision total knee arthroplasty: management and outcomes

**DOI:** 10.1186/s43019-022-00158-y

**Published:** 2022-06-20

**Authors:** Thomas Bieganowski, Daniel B. Buchalter, Vivek Singh, John J. Mercuri, Vinay K. Aggarwal, Joshua C. Rozell, Ran Schwarzkopf

**Affiliations:** 1 Division of Adult Reconstruction, Department of Orthopedic Surgery, NYU Langone Health, 301 East 17th Street, New York, NY 10003 USA; 2 Department of Orthopedic Surgery, Geisinger Health, Scranton, PA USA

**Keywords:** Knee, Revision, Arthroplasty, Bone loss, Anderson Orthopaedic Research Institute classification

## Abstract

**Background:**

Although several techniques and implants have been developed to address bone loss in revision total knee arthroplasty (rTKA), management of these defects remains challenging. This review article discusses the indications and management options of bone loss following total knee arthroplasty based on preoperative workup and intraoperative findings.

**Main text:**

Various imaging modalities are available that can be augmented with intraoperative examination to provide a clear classification of a bony defect. For this reason, the Anderson Orthopaedic Research Institute (AORI) classification is frequently used to guide treatment. The AORI provides a reliable system by which surgeons can classify lesions based on their size and involvement of surrounding structures. AORI type I defects are managed with cement with or without screws as well as impaction bone grafting. For AORI type IIA lesions, wedge or block augmentation is available. For large defects encompassing AORI type IIB and type III defects, bulk allografts, cones, sleeves, and megaprostheses can be used in conjunction with intramedullary stems.

**Conclusions:**

Treatment of bone loss in rTKA continues to evolve as different techniques and approaches have been validated through short- and mid-term follow-up. Extensive preoperative planning with imaging, accurate intraoperative evaluation of the bone loss, and comprehensive understanding of all the implant options available for the bone loss are paramount to success.

## Introduction

Patients undergoing total knee arthroplasty (TKA) are at risk of sustaining bone loss due to several etiologies including stress shielding, infection, osteolysis, mechanical bone loss due to a loose implant, or even iatrogenic loss during revision surgery [1]. Despite the development of multiple modern revision implants, the management of these bone defects remains one of the biggest challenges in revision TKA (rTKA). The goals of rTKA are similar to primary TKA: to restore the patient’s limb alignment, joint line, soft tissue balance, patellar tracking, and range of motion. Bone loss can be limited with a stable, well-positioned implant [2].

Given the increased complexity of rTKA compared with primary TKA, accurately classifying bone defects is critically important in determining treatment algorithms. Preoperatively, a myriad of radiographic techniques can be used to estimate the size of a defect, but the extent of bone loss is not fully understood until the components are removed intraoperatively and debridement of any fibrous or necrotic tissues has taken place. As a result, many classification systems based on the size, severity, and location of the defect have been created to guide treatment [3–6]. Therefore, having a detailed preoperative plan with multiple contingency plans is crucial for success. In this review article, we discuss the diagnosis, workup, and treatments of bone loss following TKA.

### Preoperative planning

Prior to evaluation with laboratory tests and/or radiographs, a comprehensive patient history involving a thorough physical examination should be completed. Query of the pain location and characteristic may help the surgeon to determine the etiology of bone loss. Assessment of inflammatory markers including an erythrocyte sedimentation rate (ESR) and C-reactive protein (CRP) level is the next step in evaluating patients presenting with pain after TKA [7]. Elevations in these levels (ESR > 30 mm/h or CRP > 1 mg/dL) should prompt aspiration of the joint to assess the cell count, differential, and fluid cultures [8].

After infection has been ruled out, there are several options for radiographic evaluation. Anteroposterior (AP) and lateral radiographs will allow the surgeon not only to infer possible causes of failure but also to assess the quality of implant fixation. Bilateral full-length standing AP radiographs can be used to characterize alignment, and oblique radiographs may better reveal osteolysis [9]. The Merchant view helps to demonstrate appropriate patellar tracking. Various techniques such as dual-energy computed tomography, magnetic resonance imaging with high bandwidth optimization, view angle tilting, multiacquisition variable-resonance image combination, and slice encoding for metal artifact correction have been developed to limit metal artifact interference and allow for better detection of bone loss, loosening, and possible infection [10–13].

### Intraoperative examination

To minimize bone loss during implant removal, it is best to mechanically disrupt the implant–bone interface circumferentially before forcibly displacing the implant with a mallet and tamp. Failure to do so can result in large sections of metaphyseal and cortical bone fracturing away with the removed implant. Special focus should be given to the less accessible undersurfaces of the implant such as the posterior femoral condyles and the posterolateral tibial plateau. Power instruments such as a sagittal saw or high-speed pencil-tip burr can be used to trace the underside of the implants. Manual instruments such as a Gigli saw wire are also reasonable options, but they may be more time consuming and technically challenging. If osteotomes are used, thin ribbon osteotomes are preferred to avoid compression of the underlying bone stock that occurs when forcing a thicker osteotome into the interface. When using a mallet and tamp or extractor, the vector of removal should be the opposite of the insertion vector. Using the incorrect vector can cause fracture of the underlying bone when buried aspects of the implants such as the femoral box or tibial keel rotate out of the host bone. After implant removal, time must be taken to carefully remove any remaining cement mantle. Cement mantle that may be deeper in the canals should be removed with specialized instruments using similar techniques to hip surgery. Also, the surgeon must be prepared to remove implants and cement using controlled osteotomies rather than uncontrolled fractures. Extensile exposures such as femoral oval windows and tibial tubercle osteotomies may sometimes be necessary.

Following implant removal and debridement of nonviable tissue, careful intraoperative evaluation is required to determine the extent of reconstruction required. Optimal results are obtained only when the joint line has been restored and physiologic range of motion through flexion and extension is recreated [14–16]. The joint line is a three-dimensional construct composed of a flexion and extension gap, each determined by different components. Accordingly, the flexion gap can be approximated by the femoral component AP size, AP translation, flexion/extension of the femoral component, and the tibial component height and polyethylene thickness. On the other hand, the extension gap is adjusted mostly through the distal femoral component position, tibial component height, and polyethylene thickness.

While imperfect and sometimes difficult to distinguish intraoperatively, anatomical landmarks must also be considered when evaluating for bone loss, whereby normal values are calculated using the joint line as a reference. One may theoretically estimate femoral bone loss by assuming that the normal distance from the lateral and medial epicondyles to the joint line is 25 mm and 30 mm, respectively. The adductor tubercle is usually 40–45 mm proximal to the joint line. Furthermore, a rough calculation of the depth of tibial bone loss begins with the presumption that the fibular head is 15 mm distal to the joint line [17].

With this in mind, rTKA can be broken down into three steps, generalized as follows: (1) reestablish the tibial platform, (2) restore the flexion gap, and (3) reconstitute the extension gap [18]. The management of bone loss plays a large role in establishing the tibial platform, flexion gap, and extension gap. Therefore, interventions must be based on preoperative planning and intraoperative estimates of bone loss.

### Classification of bone defects in revision total knee arthroplasty

There are several classification systems that have been developed to describe bone deficiencies in primary TKA and rTKA. The Dorr classification characterizes defects as being central or peripheral, and separates cases based on primary or revision procedures. While simple and straightforward, this classification system does not define the size of a defect, nor does it involve femoral defects. The nature of this system precludes it from assessing complex bone defects, and it is less helpful in rTKA [4]. The Elia and Lotke classification defines bone defects as large or small [3]. Although it provides a definition of large defects (> 1 cm in depth and > 50% of the osteotomized femur or tibia), this system is too simple to describe commonly encountered defects. Therefore, it may not be able to guide treatment nor predict clinical outcomes. The Insall classification separates bony defects into contained and uncontained [5]. Contained defects have retained their cortical rim, while uncontained defects have bone loss that lacks a cortical rim. A number of treatment options are considered in this classification system, including cementation alone, cementation or augmentation with a stemmed component, stem extensions, and block augmentation. Comprehensive definitions of the size and shape of both femoral and tibial defects are provided by this system; however, critics of this classification cite outdated descriptions of uncommon morphologies encountered with old implant designs.

The Anderson Orthopaedic Research Institute (AORI) bone defect classification is the most widely accepted scheme that allows for communication, comparison, and management recommendations [6]. The classification is summarized in Table 1. Given its consideration of both the location of the defect and the stability of the implant, the AORI classification allows for preliminary planning based on preoperative radiographs [19, 20]. However, the classification must be confirmed by intraoperative findings as there are no guidelines or tools for quantification of bone loss on preoperative imaging. Furthermore, increased bone loss occurs during implant extraction. Consequently, preoperative underestimation of bone loss is common, especially when obscured by radiopaque implants. Lesions are stratified into three ranks depending upon size, localization, and involvement of surrounding soft tissues. Type I defects have minor and contained cancellous bony defects with intact metaphyseal bone. The adequate cancellous bone around the joint line maintains implant stability. These are treated with cement, cement with screws, or impaction bone grafting. Type II defects are defined as moderate or severe cancellous and/or cortical bone defects. Type IIA defects involve one femoral condyle or tibial plateau, while type IIB defects involve both femoral condyles or tibial plateaus. Depending on the case, type IIA lesions can be managed with modular stems, wedge or block augmentation, cement with screws, or impaction bone grafting. Type III lesions compromise a major portion of the femoral condyle or tibial plateau and may also involve ligamentous or tendinous insertions. Type IIB and type III defects are treated with modular stems, bulk allografts, cones, sleeves, or megaprostheses.

**Table 1:** Anderson Orthopaedic Research Institute (AORI) rTKA bone defect classification

Type	Description
I	Minor and contained cancellous bony defects that do not affect implant stability
II	Moderate to severe cancellous and/or cortical bone defects
	IIA: one tibial plateau or femoral condyle
	IIB: both tibial plateaus or femoral condyles
III	Massive cavitary and segmental bone loss of both tibial plateaus and/or femoral condyles with/without ligament or tendon involvement

## Management of bone defects

### Cement

The indications for and applications of polymethyl methacrylate (PMMA) have evolved with the field of orthopedics. PMMA alone is indicated for contained and uncontained bone defects measuring < 5 mm in depth, such as an AORI type I defect [21]. Antibiotic-laden cement can also be used to deliver antibiotics in patients at higher risk of periprosthetic joint infection [22]. There are several instances where cement should be used with caution, however, as PMMA does not provide biologic fixation and cannot restore lost bone stock [23]. Cement is also subject to fragmentation, which may lead to prosthetic loosening [24], thermal necrosis secondary to the natural exothermic reaction involved in polymerization [25], and the potential for fat embolism [26].

To be successful with cementation, the surgeon should begin by assessing for bone defects after making freshening bony skim cuts. Given that cement must interdigitate with cancellous bone, defects should be debrided of all fibrous tissue and sclerotic bone should be roughened. Cement is strongest in compression, so any slopes or angles should be cut into step-offs. Several authors have described successful outcomes following rTKA that involved cement. Berend et al. examined 248 knees that required rTKA and received cement with or without screws from 1989 to 2010, with a mean follow-up time of 7.4 years (range 2–19.9 years) [27]. At 15 years of clinical follow-up, patients who received revision with cement alone had a higher survival probability of 0.9859. Three knees required re-revision due to unknown complications. Lotke et al. reported on 59 rTKAs that received cement fixation and were followed for an average of 7.1 years (range 5–11 years) [28]. Average clinical scores for all knees increased from 28 points preoperatively to 78 points postoperatively. Two patients required re-revision: one for infection and one for aseptic loosening after sustaining a fall.

### Cement with screws

**Fig. 1:**
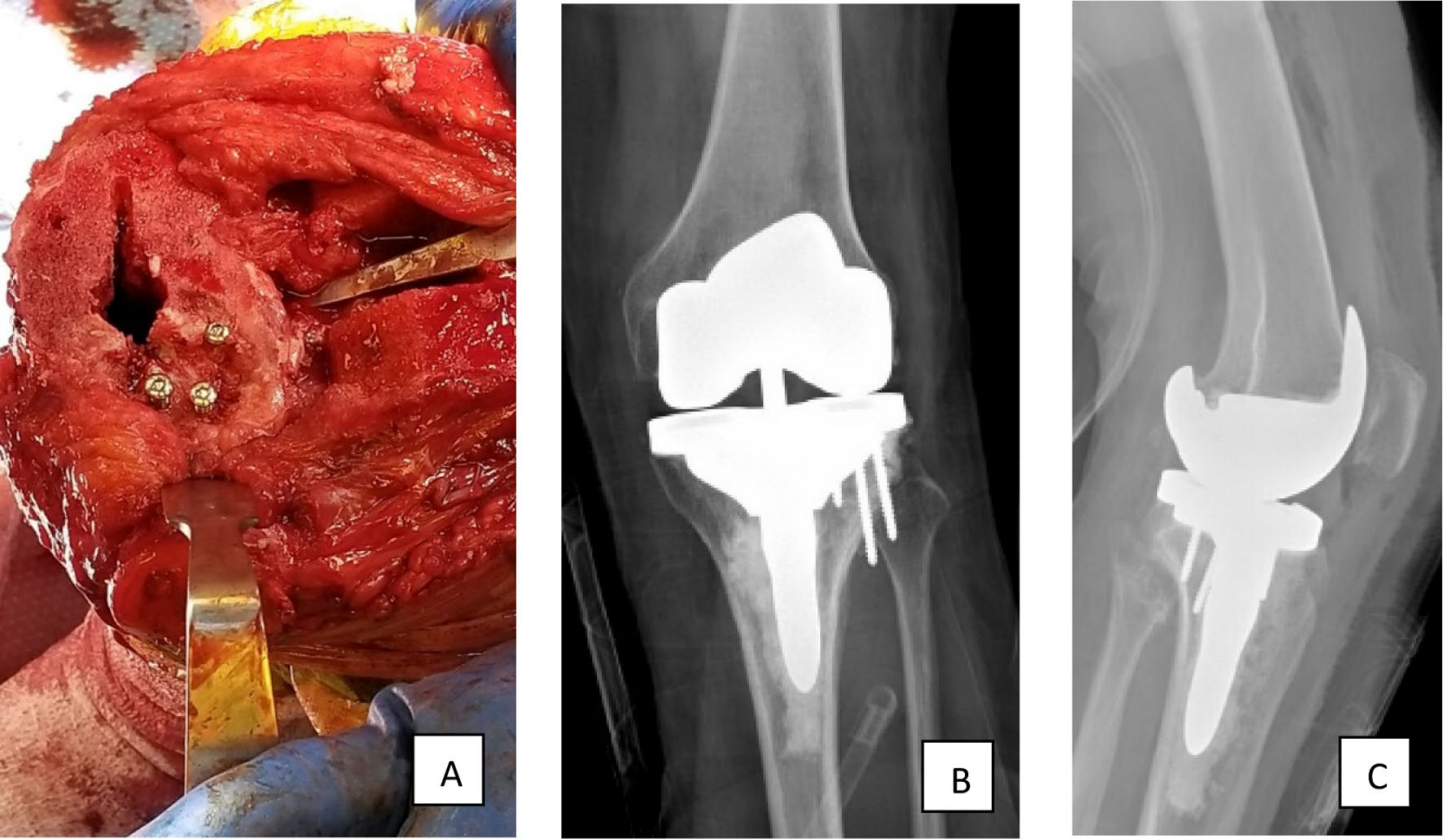
**A** Gross anatomy and **B**, **C** radiographic imaging of fixation screws with rebar technique in the tibial plateau

The indications for cement with screws are slightly broader than cement alone, and they can be used in larger AORI type I defects and type IIA defects, with recommendations including contained and uncontained defects up to 10 mm [21]. Most commonly, 3.5-mm cortical screws should be advanced into the metaphyseal defect until they engage distal bone. The length of the screws should be such that the screw heads are entirely within the defect and can be buried in the new cement mantle. Screw contact with the eventual implant should be avoided, and the defect should be filled with cement that buries the screw head immediately before implantation of the final prosthesis (Fig. 1). The combination of cement with the addition of screws has multiple advantages including enhanced strength of fixation when compared with cement alone, lower cost compared with other options, and simplification and shortening of operative times when compared with bone grafting and metal augmentation [29].

Ritter et al. followed 57 patients with tibial defects of up to 9 mm in height [30]. Although 25% of patients had nonprogressive radiolucency at the interface between bone and cement, no components failed and there was no progression of radiolucency at 7 years postoperatively.

### Modular stems

Cases of rTKA where the articular and metaphyseal bone are compromised require the addition of a stem to facilitate load transfer and distribution of stress. Modular stems can be used for a range of defect sizes including AORI type IIB and III, but also type I and IIA to bypass metaphyseal bone defects and diminish the strain at the implant–host bone interface. The method by which stems should be fixed during rTKA continues to be a matter of debate. Cemented stems are metaphyseal engaging and can be implanted with a cement restrictor to enhance interdigitation of the cement with host cancellous bone [31]. This technique provides immediate fixation with shorter stems that do not influence the final implant position. This may make removal of the implant more difficult during re-revision surgery, however, and patients who receive cemented stems are at a higher risk of stress shielding of the metaphyseal bone. Hybrid stem fixation combines cement placement at the metaphyseal implant–bone interface with a press-fit model that can engage the cortical bone of the diaphysis [32]. These implants are generally longer and provide greater stability but may need to be offset if the diaphyseal engagement causes proximal malalignment. Longer and stiffer stems may also result in end-of-stem pain in the tibial region.

Several articles have supported the use of both techniques during rTKA, making the choice of stem fixation controversial and largely dependent on surgeon preference. Kosse et al. conducted a randomized controlled trial whereby fully cemented and hybrid stems were compared using radiostereometric analysis at various time points following rTKA [33]. At 6.5 years postoperatively, 23 patients spread across both cohorts showed no significant difference in median total translation and rotation of the femoral and tibial components. Additionally, there were no significant differences between Knee Society Scores (KSS), Knee Osteoarthritis Outcome Scores, or visual analog scales for pain and satisfaction.

### Impaction bone graft

Indications for impaction morselized bone grafting in rTKA include AORI type II and III defects as well as mild contained and uncontained bone defects that involve a depth of < 50% of the femoral condyle or tibial plateau [21, 34]. Whether using autograft or donor allograft, bone chips should be as large as practical, up to 5 mm in diameter, to ensure stability. To use a morselized graft in a contained defect, the surgeon must first debride the defects to remove all fibrous tissue. There must be a vascularized, bleeding bed of host bone, and sclerotic bone should be burred away to facilitate this. Then, a finely ground morselized bone graft is tightly packed into the defect, and the final components can be implanted. If a more stable bone grafting construct is needed, metal wire mesh can be utilized. This technique allows surgeons to mold and contain the graft based on patient-specific requirements. Although preferred in younger patients who may require further revision, the impaction force required to set the graft cannot be quantified, making the integrity of the construct uncertain. Furthermore, bone graft takes time to incorporate and thus may not be as immediately stable as cement augmentation [35].

The use of impaction bone grafting is an established technique that has classically been described for the management of bone loss in rTKA. Hanna et al. retrospectively reviewed 56 patient who underwent rTKA with long-stemmed components that were reinforced with morselized bone graft from 1999 to 2006 [36]. All patients had a minimum follow-up time of 4 years. At 10 years postoperatively, cumulative prosthesis survival was 98% and mean Oxford Knee Scores (OKS) improved from 21 to 41 at date of last follow-up. Five patients required reoperation for lateral collateral ligament reconstruction, spacer exchange, patellar baja exploration, Roux–Goldthwait procedure, and two-stage revision to a knee fusion secondary to infection.

Bedard et al. examined 35 rTKAs that involved the use of diaphyseal impaction bone grafting from 2005 to 2016 [37]. Patients had a mean follow-up time of 4 years and 100% survival free from revision of the impaction grafting construct due to aseptic loosening at 4 years. All unrevised knees exhibited incorporation of the impaction bone graft radiographically. A total of six patients required re-revision secondary to infection (*n* = 4) and periprosthetic fracture (*n* = 2).

### Augments

**Fig. 2:**
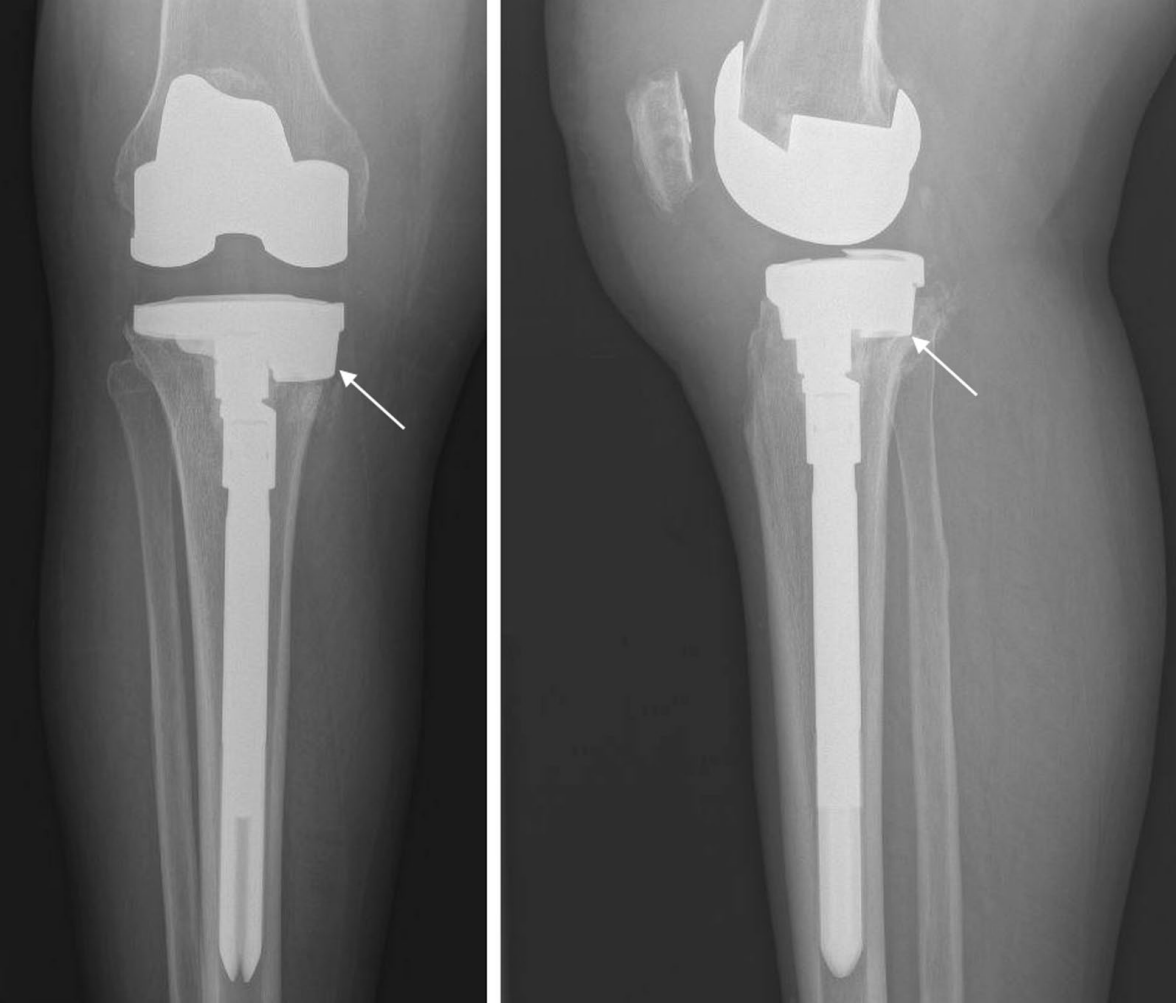
AP (left) and lateral (right) radiographic imaging demonstrating a medial tibial augment (arrow) and hybrid fixation with metaphyseal cementing, an offset connector, and a cementless tibial stem

Augments have become highly specialized and are classified based on the involvement of tibial (Fig. 2) or femoral components, but are generally reserved for patients with AORI type IIA and IIB defects. Most augments are made from tantalum or titanium, which not only are biocompatible but also have thrombogenic potential to encourage hematoma formation and bone healing [38]. The field of three-dimensional (3D) printing has also revolutionized the way complicated orthopedic problems are managed. In a cadaveric study, Dion et al. demonstrated that 3D-printed titanium augments achieved significantly less micromotion than standard fully cemented stems in fresh-frozen tibias status post removal of the primary TKA construct [39]. In the coming years, patients suffering from large bony defects may be reconstructed with 3D-printed augments that are tailored to their specific anatomy.

### Tibial augments

Tibial augmentation can be performed with modular metal wedges or blocks and is considered when defects encompass 5–20 mm of depth, particularly if these defects fail to support at least 25% of the tibial baseplate. Although the decision to use wedge or block augmentation is case specific, block augments generally have a lower rate of implant loosening and are more stable than wedges due to lower shear forces [40]. On the other hand, block augments are expensive and require substantial bone removal to accommodate the insert [41].

### Femoral augments

Femoral bone deficiency may be treated with block-shaped augments with varying thicknesses ranging from 5 to 15 mm [42]. Femoral augments are designed for the medial and lateral condyles both distally and posteriorly. Posterior femoral augments are of particular benefit as they promote restoration of the femoral component in the anteroposterior dimension and alter the flexion gap, which may address extension–flexion mismatch. Placing a femoral augment posterolaterally will also prevent internal rotation of the femoral component.

Mid-term results are available for patients who received augments during rTKA. Stambough et al. examined seven patients from 2006 to 2014 with uncontained, unicondylar tibial bone defects that had an excessive loss of supportive cortices in the metadiaphyseal region after undergoing TKA [43]. These patients were treated with highly porous metal acetabular wedge augments and had a minimum 3-year follow-up with an average of 5 years (range 3–12 years). All wedge augments showed no clinical evidence of failure at last follow-up, and no patients required re-revision. One patient died from septicemia 15 months after revision surgery due to unknown causes. Crawford et al. analyzed the outcomes of 274 knees that underwent rTKA with a modular system composed of cobalt chrome or titanium from 2005 to 2013 [44]. Patients had a minimum of 2 years of follow-up and a mean follow-up of 6 years (range 2–11 years). Clinical KSS rose from 45 to 79 (*p* < 0.0001), and function scores from 46 to 56 (*p* < 0.0001). Since the initial revision, there have been 25 aseptic revisions, 15 of which were secondary to aseptic loosening.

### Bulk allograft

Bulk structural allograft is typically indicated for large defects (AORI type II and III) that exceed the dimensions of metal augments. The femoral head is a common allograft choice. The defect and the allograft must be shaped to fit together. As with morselized grafting, bulk allograft should contact vascularized, nonsclerotic host bone. The grafts should be fixed in place with screws, and metal-on-metal contact between screws and the implants should be avoided. Bulk allograft offers the potential benefit of rebuilding bone stock in a young patient. However, they have a risk of nonunion, resorption, or collapse leading to structural instability [45]. In such cases, the addition of a stem may be warranted to protect the graft from excessive load. Without a stem in place, the load experienced by the knee joint will expose the cancellous bone to weight greater than its ultimate strength [46].

There is copious literature demonstrating the benefit of structural allograft in rTKA for patients with severe bone defects. Chun et al. assessed 27 patients who underwent rTKA from 1997 to 2003 with a fresh-frozen femoral head allograft and a diaphyseal-engaging stem [47]. At a median follow-up of 107 months (range 96–157 months), the mean range of motion increased from 71° to 113°, and the mean Hospital for Special Surgery knee score improved from 46 to 83. Failure occurred in only one knee secondary to infection; otherwise, 26 out of 27 knees demonstrated union with no evidence of collapse at an average of 7 months postoperatively.

Sandiford et al. compared postoperative outcomes in 45 patients undergoing rTKA with femoral head allograft (*n* = 30) versus trabecular metal augments (*n* = 15) between 2002 and 2008 [48]. There was a mean follow-up time of 9 years, and no patient was lost to follow-up. They found no significant differences in mean OKS and Western Ontario and McMaster Universities Osteoarthritis Index Scores. Five-year survivorship was higher (93% versus 91%) in the allograft group, and they found no significant differences in surgical complications. One patient in the allograft group sustained a periprosthetic fracture.

### Cones

**Fig. 3:**
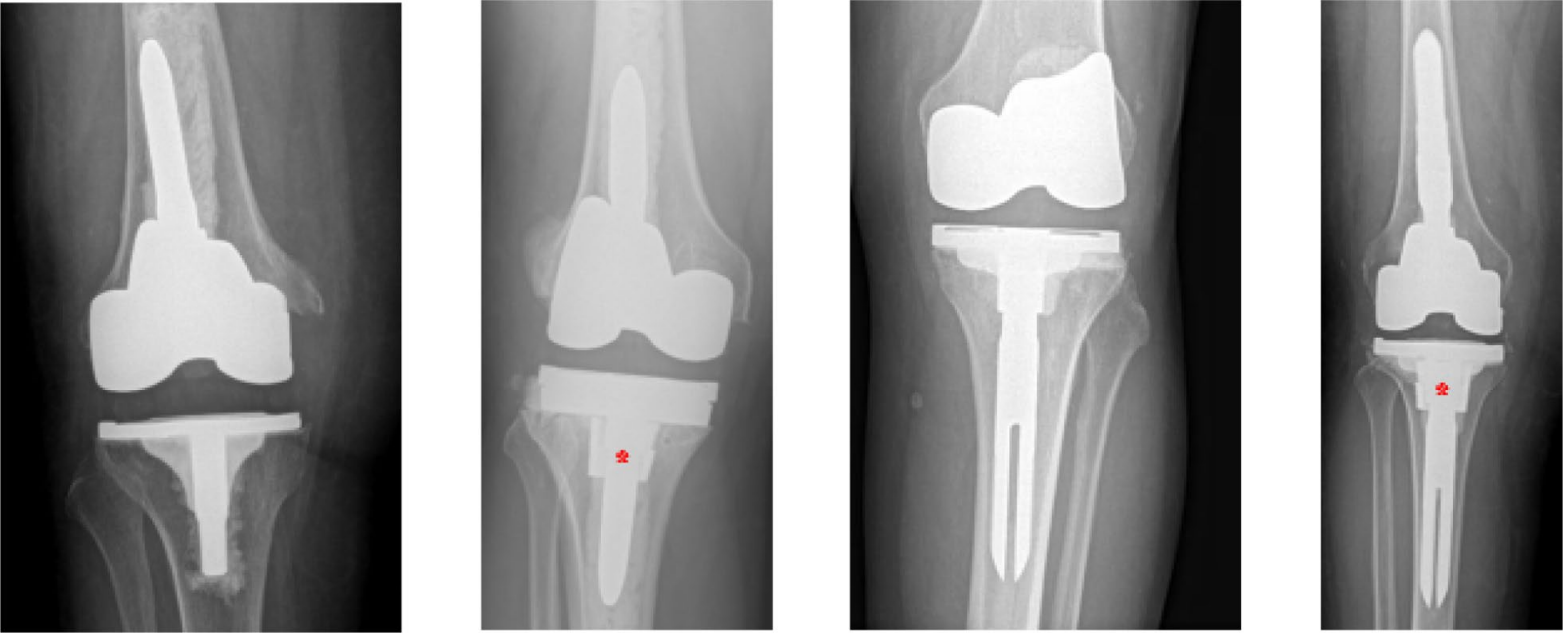
Radiographic imaging of cemented and hybrid stem technique, with and without cone augmentation. From left to right: cemented without cone; cemented with cone; hybrid without cone, hybrid with cone. *Indicates the presence of a cone

Cones are usually reserved for AORI type II and III defects where the metaphyseal bone is largely absent [49, 50]. Recently, tantalum and titanium have been used due to their high biocompatibility, porosity, and osteoconductive potential, as well as their similar modulus of elasticity to cortical bone [51]. The surgeon must begin by removing previous implants, cement, membrane, and all nonviable tissue. This is followed by reaming of the femoral and tibial canals, which provides a foundation for bone preparation with broaches. Further accommodations for the cone can then be made by using a high-speed burr to shape the metaphysis as needed, skim cuts can be made, and augments added as needed. Trial components can then be placed, and a metal cone chosen that fits the defect. The stem can then be cemented in place through the cone’s center. Additionally, fixation may be achieved with a hybrid technique that utilizes a cementless press-fit stem (Fig. 3). No superiority of either fixation method has been found, and ultimately, metaphyseal cones represent an opportunity to achieve mid- to long-term success, even with severe bone loss [46]. The advent of cone use in rTKAs has made the procedure less technically demanding and more streamlined for surgeons who may have once used structural bone grafts. Despite their known advantages, however, cones often require that the surgeon remove bone before implantation to accommodate the insert.

Many studies have examined the outcomes of rTKA following cone placement. Remily et al. performed a retrospective analysis of 54 patients who underwent an rTKA from 2015 to 2017 for an AORI type II or III defect and were treated with a 3D-printed femoral or tibial porous titanium metaphyseal cone [52]. Patients had a minimum 2-year follow-up with a mean follow-up of 29.9 months (range 24–42 months). Radiographic analysis revealed that 51 of the cones were well fixed without evidence of loosening or migration. Additionally, mean postoperative KSS were significantly higher when compared with preoperative scores (80.4 versus 52.0, *p* > 0.001). Survivorship was 98.5%, 88.2%, and 77.9% for aseptic loosening, cone revision for any reason, and reoperation of the knee for any reason, respectively. One patient who received both tibial and femoral cones underwent re-revision secondary to aseptic loosening of the femoral cone, while another patient who also received both tibial and femoral cones developed a prosthetic joint infection requiring two-stage revision. Lastly, five patients with either a femoral or tibial cone developed a prosthetic joint infection requiring two-stage revision.

Chalmers et al. retrospectively examined 163 patients who underwent rTKA with a porous tibial cone from 2016 to 2018 [53]. The mean age was 67 years, and a minimum follow-up duration of 2 years was established with a mean follow-up of 2.5 years (range 2–4 years). Their reports include 100% survivorship free from re-revision due to aseptic loosening, 96% free from any nonmodular revision, and 86% free from any reoperation at 2 years. In total, there were 23 reoperations, including the 6 nonmodular re-revisions requiring cone removal all secondary to PJI.

Ohlmeier et al. examined the clinical outcomes of calcium-phosphate-coated tibial cones by retrospectively analyzing 52 patients requiring rTKA for AORI type IIA (17), type IIB (14), and type III (21) bone defects [54]. Patients were selected from January 2016 to December 2017 with a mean follow-up of 22 months. Mean OKS postoperatively was 28.6 points, and 22 knees showed appropriate radiographic positioning of the cones at a mean follow-up of 16.8 months. Three patients had bone–cement interface radiolucencies, and three patients had heterotopic ossifications. Two patients required re-revision secondary to infection, and two more patients required re-revision due to aseptic reasons, involving patellar dislocation in one case and loosening of the tibial component including the tibial cone in the other.

### Sleeves

Currently, titanium metal sleeves in rTKA are indicated in cases of large contained AORI type IIA, IIB, or III bone defects. While sleeves serve the same purpose as cones, sleeves are implant specific, unitized to the stem, and inserted with the entire prosthesis as one unit. Despite these nuances, the placement of sleeves is similar to that of tibial cones with reaming of the medullary canal and subsequent sizing of the sleeve. The construct is then built with the sleeve attached to the stem and femoral or tibial component, and the prosthesis is impacted and may be cemented or uncemented. In such cases, metaphyseal sleeves may provide the added benefit of creating a supportive structure for reconstruction when there is compromise of the metaphysis. Similar to cones, tibial sleeves are expensive and can be difficult to extract after osteointegration [55, 56].

Several authors have outlined the benefit that sleeves can provide during rTKA with severe bone loss. Panesar et al. explored the outcomes associated with 99 rTKAs of rotating hinge knee prostheses performed with uncemented metaphyseal sleeves from 2002 to 2018 [57]. The mean follow-up time was 7 years (range 3.6–11.6 years). Sixty-seven cases were for aseptic revisions, while 32 were in the setting of infection. At the latest follow-up, OKS had risen by 15 points with survivorship of 81%. Twenty-six patients required re-revision secondary to infection (10), patella resurfacing (5), failure of bony ingrowth (2), and fracture (1). Algarni et al. investigated outcomes associated with rTKA by reviewing 52 knees that required a metaphyseal sleeve with a cementless tibial or femoral stem from 2012 to 2018 [58]. The mean follow-up time was 4.1 years (range 2.0–7.5 years) with a minimum follow-up of 2 years. Following rTKA, the range of motion improved by 17° on average (*p* = 0.19) and KSS increased by just under 28 points (*p* < 0.001). Aseptic loosening survivorship and overall survivorships were 100% and 96.3%, respectively, with only one case of sustained fracture and reoperation.

### Megaprostheses

**Fig. 4:**
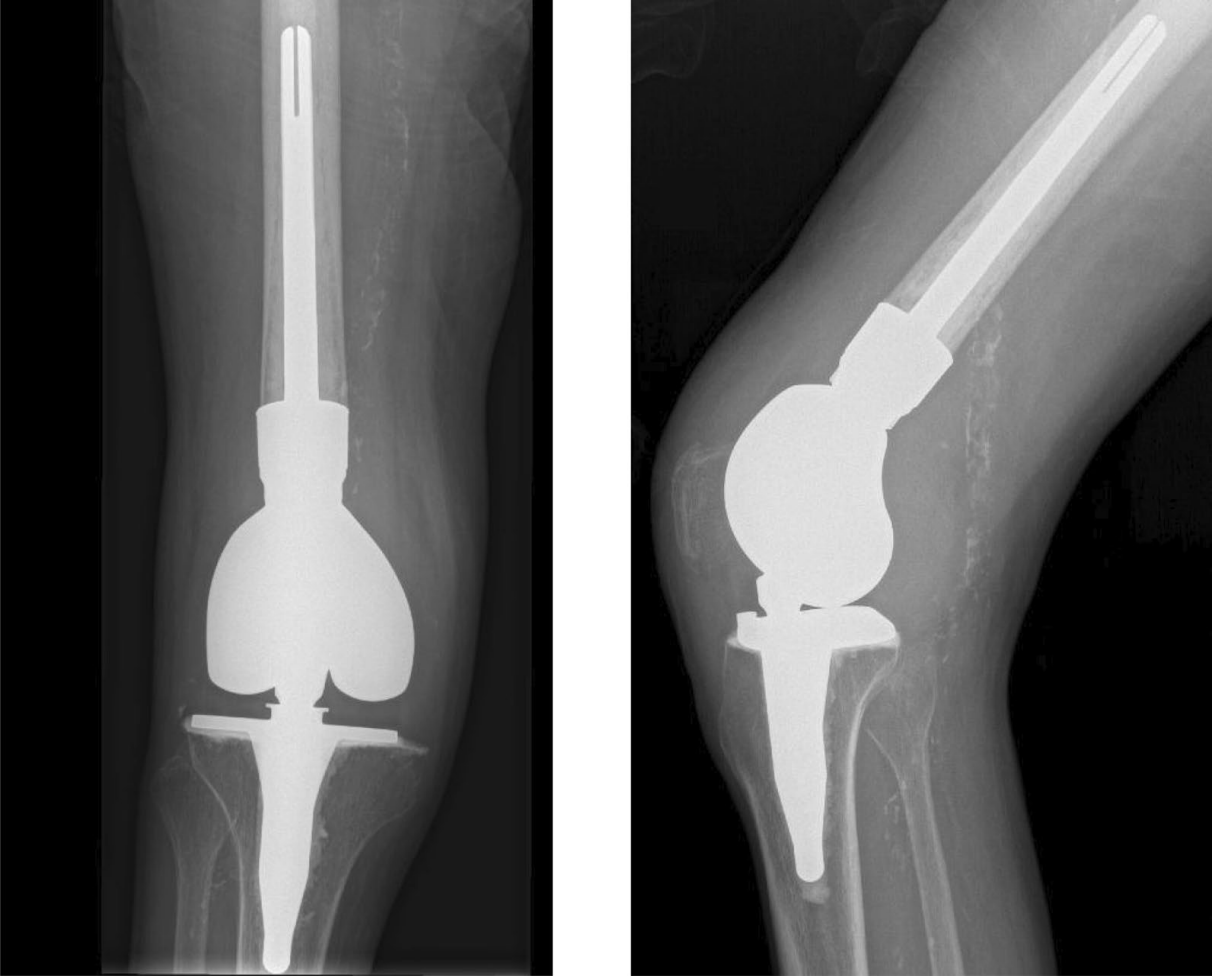
AP (left) and lateral (right) radiographic imaging of a distal femoral replacement megaprosthesis

Traditionally reserved for knee reconstruction cases following tumor resection, megaprostheses may also be used in patients with severe bone loss, such as an uncontained AORI type III defect [59, 60]. Typically encountered following chronic infection or multiple surgeries, megaprostheses are usually reserved for patients where replacement of the distal femur (Fig. 4) or proximal tibia is required [61]. In cases of non-reconstructible bone loss where a limb-salvage procedure is required, megaprostheses represent a viable solution with a relatively rapid rehabilitation period that can ultimately provide a stable construct for the patient [62, 63]. Introduction of a rotating hinge platform along with the use of modular endoprosthesis instead of custom-made implants have decreased failure rates and improved availability and versatility [64–66]. Surgeons opting to use megaprosthesis should be aware of the technical challenges generally encountered when using these implants. The complications associated with megaprostheses include patellar maltracking and component malrotation as well as the magnitude of the revision if the prosthesis gets infected [67].

Holl et al. looked at the outcomes of 21 knees in 20 patients who underwent rTKA with a megaprosthesis between 2000 and 2010 [61]. The average patient age was 73 years, and all 20 patients had nononcologic indications for megaprosthesis, including periprosthetic infection (5), fracture (9), nonunion (5), and aseptic loosening (2). At a mean follow-up time of 34 months (range 10–84 months), KSS had significantly improved from 43 ± 15 to 68 ± 16.8 (*p* < 0.05). Notably, 11 patients suffered complications involving infection (6), fracture (2), and aseptic loosening (2) as well as one patient who had persistent wound healing problems.

Grammatopoulos et al. examined the outcomes of 80 knees in 79 patients who underwent rTKA with a modular femoral endoprosthesis from 2005 to 2014 [68]. Patients had a mean follow-up of 5 years (range 0.1–11.5 years) and an average age of 69 years. Overall survival at 5 years was 87%, and a “worst-case scenario” analysis determined a 5-year survival of 74%. Although 25 patients experienced a complication following surgery and 18 patients required further surgery, limb salvage was achieved in all patients.

Vertesich et al. retrospectively reviewed 30 patients requiring rTKA with distal femoral reconstruction involving a modular megaprostheses from 1997 to 2017 [67]. The mean age of their cohort was 74.38 years, and mean follow-up time was 54.15 months (range 1–240 months). Revision-free survival was 74.8% at 1 year, 62.5% at 3 years, and 40.9% at 10 years postoperatively. Of the total number of patients included, 16 patients had at least one complication involving soft-tissue failure (*n* = 3), aseptic loosening (*n* = 4), and structural failure (*n* = 1) requiring revision surgery. The remaining eight patients suffered infection, three of whom required revision surgery.

## Conclusions

As the demand for TKA continues to rise in the coming decades, surgeons will be tasked with improving the techniques and hardware employed to manage revisions. Bone loss during rTKA represents a complex issue that must take into consideration all facets of underlying pathology to avoid exposing patients to further revision. Extensive preoperative planning with imaging, accurate intraoperative evaluation of the bone loss, and comprehensive understanding of all the implant options available for the bone loss are paramount to success.

## Data Availability

Not applicable.

## Abbreviations

rTKA: Revision total knee arthroplasty
AORI: Anderson Orthopaedic Research Institute
TKA: Total knee arthroplasty
ESR: Erythrocyte sedimentation rate
CRP: C-reactive protein
AP: Anteroposterior
PMMA: Polymethyl methacrylate
KSS: Knee Society Scores
OKS: Oxford Knee Score
